# Modified iPOND revealed the role of mutant p53 in promoting helicase function and telomere maintenance

**DOI:** 10.18632/aging.205117

**Published:** 2023-10-12

**Authors:** Qianqian Wang, Kailong Hou, Jun Yang, Haili Li, Cui Li, Yanduo Zhang, Jie Tian, Chuanbiao Li, Bing Guo, Shuting Jia, Ying Luo

**Affiliations:** 1 Department of Pathophysiology, School of Basic Medicine, Guizhou Medical University, Guiyang 550025, Guizhou, China; 2 Lab of Molecular Genetics of Aging and Tumor, Medical School, Kunming University of Science and Technology, Kunming 650500, Yunnan Province, China; 3 Department of Human Anatomy, School of Basic Medicine, Shandong First Medical University and Shandong Academy of Medical Sciences, Jinan 250117, Shandong, China; 4 Institute of Molecular Physiology, Shenzhen Bay Laboratory, Shenzhen 518132, Guangdong, China

**Keywords:** p53 mutation, DNA replication stress, DNA helicase, G-quadruplex structure, telomere

## Abstract

The G-rich DNA, such as telomere, tends to form G-quadruplex (G4) structure, which slows down the replication fork progression, induces replication stress, and becomes the chromosome fragile sites. Here we described a molecular strategy that cells developed to overcome the DNA replication stress via DNA helicase regulation.

The p53N236S (p53S) mutation has been found in the Werner syndrome mouse embryo fibroblast (MEFs) escaped from senescence, could be the driving force for cell escaping senescence. We revealed that the p53S could transcriptionally up-regulate DNA helicases expression, including Wrn, Blm, Timeless, Ddx, Mcm, Gins, Fanc, as well as telomere specific proteins Terf1, Pot1, through which p53S promoted the unwinding of G4 structures, and protected the cells from DNA replication stress induced by G4 stabilizer. By modified iPOND (isolation of proteins on nascent DNA) assay and telomere assay, we demonstrated that the p53S could promote the recruitment of those helicases to the DNA replication forks, facilitated the maintenance of telomere, and prevent the telomere dysfunction induced by G4 stabilizer. Interestingly, we did not observe the function of promoting G4 resolving and facilitating telomere lengthening in the cells with Li-Fraumeni Syndrome mutation-p53R172H (p53H), which suggests that this is the specific gain of function for p53S.

Together our data suggest that the p53S could gain the new function of releasing the replication stress via regulating the helicase function and G4 structure, which benefits telomere lengthening. This strategy could be applied to the treatment of diseases caused by telomere replication stress.

## INTRODUCTION

The fidelity of DNA replication is essential for maintaining intact genetic information and genome stability. The progression of DNA replication is highly dependent on the DNA structures. The chromosome fragile sites, telomere, centromere, and rDNA sequences are composed of G-quadruplex (G4) structures, hairpins, triplexes, etc. These complicated DNA secondary structures might slow down the progression of DNA replication forks, and become the potential obstacles for DNA replication [1].

The essential function of helicase is to fire replication origins and unwind the secondary DNA structures, ensure the smooth progression of DNA replication. The defect of helicase function due to genetic mutation or environmental toxic factors might result in the DNA replication stress, manifest as stalled replication forks, or even collapsed replication forks, which further activate DNA damage response pathways [2]. The cellular strategy of dealing with DNA replication stress could prevent the progress of aging or tumorigenesis.

DNA helicase is a super family of enzymes that could hydrolyze ATP and use the energy to unwind DNA double helix. The Mcm family proteins are key helicases in regulating DNA replication. At the replication origin sites, the origin recognition complex (ORC) and Cdc6 recruit helicase Cdt1and Mcm proteins to the DNA, and form the pre-replicative complex (pre-RC). This process enable the Mcm2-7 form hexamer at the double strand DNA, which is the key complex in the origination, elongation, and progression of DNA replication [3]. The following form of Cdc45/Gins/Mcm2–7 (CMG) complex is essential for initiating the replication process [4]. Ddx11, Rtel1, and XPD/ERCC2 are helicases of SF2 family. The Ddx11 could resolve fork DNA and G4 DNA structures, and thus endow the cells with resistance to the toxic reagents that stabilize G4 structure [5, 6].

Once the DNA double helix has unwound and formed a replication fork, the single stranded DNA fork needs to be protected by fork protection complex (FPC), composed of Timeless, Tipin, Claspin and And-1 [7]. The Timeless protein is involved in regulation of circadian rhythm, DNA repair, and telomere maintenance, thus is believed to be an anti-aging protein [8]. Recent study has revealed that the Timeless-Tipin and Claspin could facilitate the binding of DNA polymerase E (PolE) and CMG (Cdc45-Mcm-Gins) to the replicated DNA. It is very interesting that during the DNA replication, the C terminal myb-like domain of Timeless could recognize and bind to G4 structure and recruit Ddx11 and PARP to G4 DNA, thus facilitate the resolving of G4 structure [9].

Telomere, a special type of repetitive genomic DNA, has a unique T-loop structure that is rich in G4, creating a physiological challenge for DNA replication progress. To prevent the replication stress induced by telomere replication, a set of proteins are assigned to the telomere DNA sites.

The Wrn, Blm, and Recql4 belong to the RecQ family of DNA helicase, and are widely involved in the process of DNA replication, DNA recombination, DNA repair, and telomere elongation, thus are essential for the maintenance of genome stability [10]. It has been shown that the Wrn protein could unwind the G4 DNA structures, which is the major bottle neck for telomere lengthening, and Wrn is essential for the lagging strand sythesis of telomere DNA replication [11]. The Blm protein could also unwind the G4 DNA structures, and is important for the leading strand sythesis of telomere DNA replication [12]. Other than this, the Blm protein could resolve other secondary DNA structure, such as Holiday junction, thus is important for the regulation of DNA replication stress. The Recql4 protein is involved in the repair of DNA double strand breaks, as well as the telomere DNA maintenance [13].

Fanconi anemia family proteins are also very important in regulating telomere DNA replication [14], and it has been shown that Fancd2 could regulate the stability and assemble of Blm protein complex on the replicated DNA, and Fancd2 and Blm cooperate to promote restart of stalled replication forks [15].

The genetic defect of DNA helicase causes progeroid syndromes, such as Werner Syndrome (caused by Wrn mutation), Bloom Syndrome (caused by Blm mutation), Rothmund-Thomson Syndrome (caused by Recql4 mutation), Warsaw Breakage Syndrome (caused by Ddx11 or Timeless mutation). Werner syndrome (WS) is an autosomal recessive disease that is characterized by premature atherosclerosis, ischemia heart disease, osteoporosis, cataracts, type 2 diabetes, sarcoma predisposition, etc., and the average lifespan of WS patients is around 45 years [16]. The molecular features of WS include chromosome instability, accelerated telomere erosion, as well as high level of DNA replication stress, thus providing a good model in studying the regulation of replication stress and aging [17]. The Werner syndrome mouse model has been established and used to study the aging phenotypes of progeroid diseases [18, 19]. By using the senescent cells derived from Werner syndrome mouse, we tried to dissect the key molecules that drove the senescent cells re-entering into cell cycle. In three independent cell lines spontaneously escaped from senescence, we found a single point mutation of the p53 gene which causes one single amino acid change of N236S in mouse p53 protein (refer as p53S later). We found that the p53S could reduce the DNA damage responses caused by the overexpression of oncogenic H-Ras, thus reduced the oncogene induced senescence (OIS), and facilitate oncogene-induced tumorigenesis [20]. However, it is a rare mutation in clinical human tumors. What is the biological significance of this p53S mutant?

By ChIP-chip and RNA-seq, we analyzed the transcription and expression profile of p53S, and we revealed the new aspects of gain-of-function for mutant p53. We found that p53S gain the function of regulation of G2M checkpoint, mitotic spindle process, as well as the DNA repair and spermatogenesis [21]. From the ChIP-chip and RNA-seq data, we also noticed that p53S might regulate the DNA replication process via regulating the expression of DNA helicases. To further understand the role of p53S in regulating DNA replication and cell cycle progression, here we applied iPOND (isolation of proteins on nascent DNA) technique to compare the changes of replication fork binding proteins in p53S. The iPOND technique first marks the newly synthesized DNA strand by incorporating the thymidine analogue EdU (5-ethynyl-2 '-deoxyuridine). Then the protein-DNA are cross-linked by formaldehyde and the EdU was covalently linked to biotin by Click reaction. The avidin magnetic beads are used to pull down the DNA-protein complex, and the proteins on the newly synthesized DNA can be analyzed by Western blot. iPOND technology enables us to identify the proteins existing on the newly synthesized DNA (replication fork) with high temporal and spatial resolution [22–24]. To improve the resolution of high molecular weight proteins, we modified iPOND technique and applied to dissect the role of p53S in regulating DNA replication, as well as in the telomere maintenance.

## RESULTS

### The p53S up-regulated the expression of DNA helicases and DNA fork protection complex proteins

**Figure 1:**
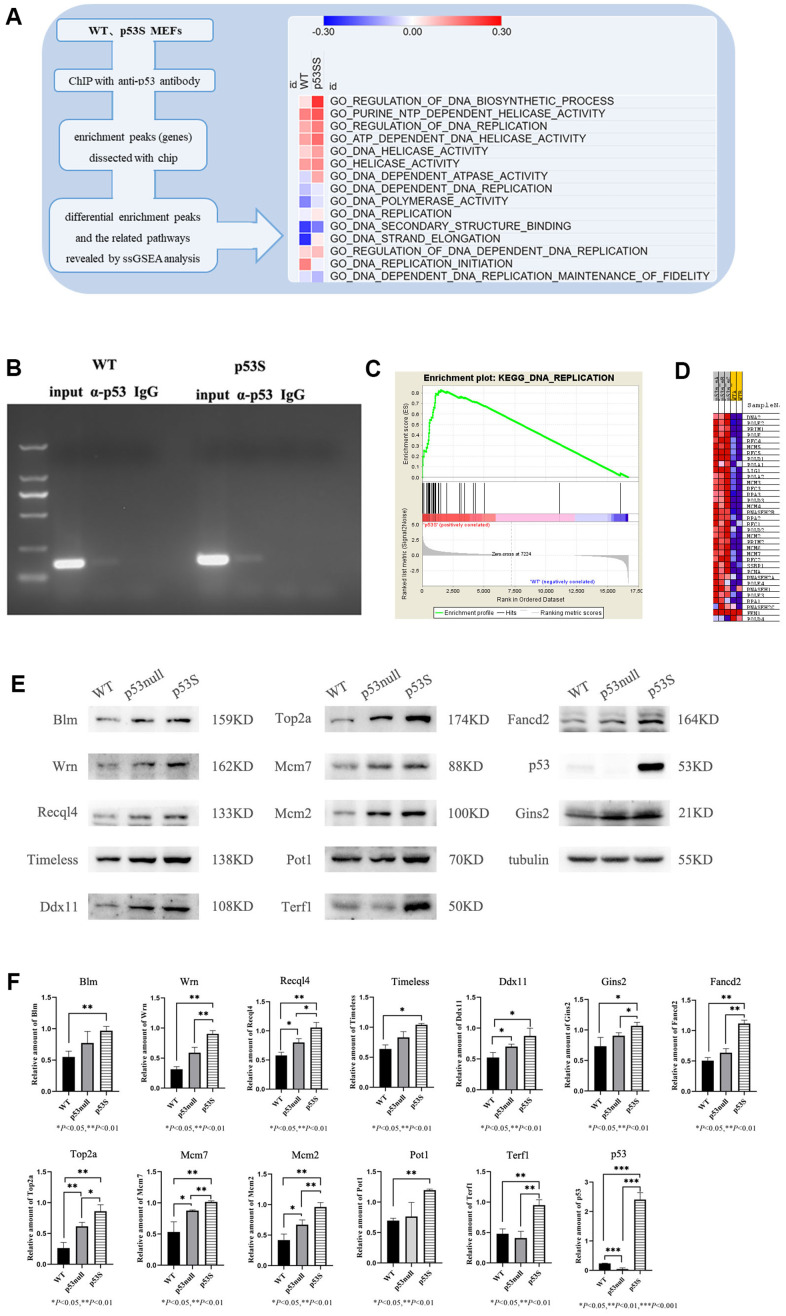
**The p53S up-regulated the expression of DNA helicases and DNA fork protection complex proteins.** (**A**) The schematic plot of the ChIP-chip assay and ssGSEA heatmap for p53S regulated pathways. The DNA replication pathways and DNA helicase pathways were up-regulated by p53S. (**B**) The binding of p53S to Mcm2 gene promoter was confirmed by the ChIP assay and semi-quantitative PCR. Both wild type p53 (left) and p53S (right) could bind to Mcm2 gene promoters with a little more abundance in p53S group. (**C**, **D**) The GSEA analysis of transcriptional enrichment in p53S cells comparing with WT cells. The enrichment plot displayed good enrichment signal in DNA replication pathways (**C**), the specific enrichment of individual genes is shown in (**D**). (**E**) The confirmation of protein expression regulation revealed by GSEA analysis. The DNA helicases, as well as telomere related proteins were up-regulated in p53S, comparing with p53null and WT cells. (**F**) The quantification of protein expression level in (**E**).

By using the mouse embryo fibroblasts (MEFs) generated from the homozygous *p53^S/S^* mice (p53S), with the control of wild type (WT) MEFs, we utilized anti-p53 antibody to perform ChIP-chip assay [21]. When we analyzed the data by single sample gene set enrichment analysis (ssGSEA), we found that p53S could transcriptionally up-regulate the DNA replication process, probably through up-regulating the activity of DNA helicases (Figure 1A), the genes involved in this regulation included Mcm2, Gins2 etc. (supplementary Figure 1). Further ChIP assay combined with semi-quantitative PCR revealed that both wild type p53 and p53S could bind to Mcm2 gene promoters, while the p53S showed higher affinity than wild type p53 (Figure 1B). To understand the expressional consequence of this regulation, we further analyzed the RNA-seq data by gene set enrichment analysis (GSEA). We found that compare to the wild type MEFs, the p53S MEFs expressed higher level of DNA replication related genes (Figure 1C), including DNA polymerases (PolD, PolE, PolA, etc.), DNA helicases (Mcm5, Mcm3, Mcm4, Mcm2, Mcm6, Mcm7, etc.), replication factors (RFC 4, RFC 5, RFC 3, RFC 1, etc.), single-stranded DNA binding proteins (RPA3, RPA2, RPA1, SSBP1, etc.) (Figure 1D). Together these data suggest that p53S transcriptionally up-regulates the expression of DNA replication related genes, especially DNA helicases.

To understand whether this function is simply due to the loss of wild type p53 function (like p53null), or the gain of function of p53S, we compared the DNA helicase protein levels in WT, p53null (*p53^-/-^*), and p53S MEFs. Consistent with the RNA-seq data, the Western blot results showed that DNA helicases and DNA replication proteins, such as Wrn, Blm, Recql4, Timeless, Ddx11, Gins2, Fancd2, Mcm2, Mcm7, Top2a were up-regulated in both p53null and p53S comparing to WT cells. Compared with the p53null cells, these proteins were further up-regulated in p53S cells. Interestingly, telomere regulated proteins Pot1 and Terf1 were also up-regulated in p53S comparing to p53null and WT cells (Figure 1E, 1F). Together, these data suggest that the p53S transcriptionally up-regulates the DNA replication proteins, especially DNA helicases, which may promote the DNA replication process and counteract the DNA replication stress. Since it is the transcriptional regulation, this effect is most likely due to the gain of function of p53S.

### The p53S reduced the presence of G4 structure and endowed the cellular resistance to DNA replication stress induced by G4 stabilizer

**Figure 2:**
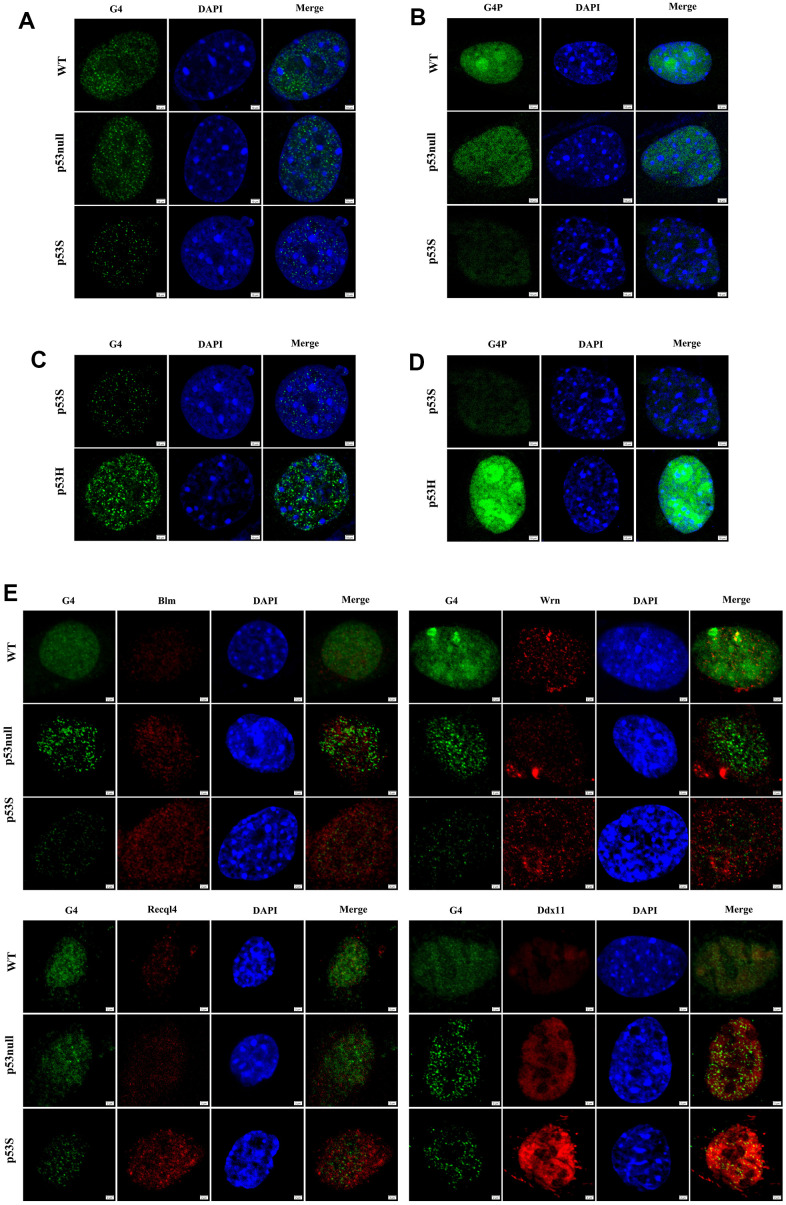
**The p53S reduced the presence of G4 structure by promoting the DNA helicase expression and colocalization with G4.** (**A**) Detected by immunofluorescence with G4 antibody, the presence of G4 structure decreased in p53S cells comparing with p53null and WT cells, suggesting a smoother unwinding of G4 structure. (**B**) Detected by specific G4 binding protein G4P labeled with GFP, the presence of G4 structure decreased in p53S cells comparing with p53null and WT cells. (**C**) The comparison of the G4 content between p53S and p53H cells by immunofluorescence with G4 antibody. (**D**) The comparison of the G4 content between p53S and p53H cells by specific G4 binding protein G4P. (**E**) The p53S promoted the expression and colocalization of DNA helicases with G4 structure.

To further investigate the cellular consequence of the up-regulation of DNA helicases in p53S cells, we compared the DNA replication process in WT, p53null, and p53S cells. Since G4 structure is known to be the major obstacle for the progression of DNA replication forks, we test the presence of G4 structures in three cell types. By using specific G4 antibody, it showed that the p53S cells had much less G4 structures than that of WT and p53null cells (Figure 2A). To further confirm the differential presence of G4 structure in these cells, we exogenously expressed a specific G4 binding proteins (G4P) in these cells by transient transfection of pNLS-G4P-IRES2-EGFP plasmids [25], and the nuclear GFP fluorescence was used to estimate the amount of G4 structure. The results illuminated that the p53S cells showed much less G4 binding proteins than that of WT and p53null cells (Figure 2B). Together these data strongly support that the G4 structures were unwound smoothly during DNA replication in p53S cells, which might be due to the up-regulation of DNA helicases by p53S.

Since the effect of unwinding G4 structure was only observed in p53S cells, not in p53null cells (Figure 2A, 2B), we confirmed that this effect is due to the gain of function of p53S. To further test whether this effect is specific to p53S function, we utilized the MEFs derived from the mouse with a p53 hot spot mutation-p53R172H (p53R175H in human, refer to as p53H) [26] to compare with p53S. By applying G4 antibody (Figure 2C) and G4P (Figure 2D) to p53S and p53H MEFs, we found that the p53H MEFs present much more G4 structures than p53S. These data suggest that the function of promoting G4 structure unwinding may be the specific gain of function for p53S mutation.

Given the idea that the unwinding of G4 structure might due to the elevated helicase level, we then performed double immunofluorescence staining to investigate the colocalization of G4 structure and DNA helicase protein. The data revealed that in the nuclei of all three cells, helicase Blm, Wrn, Recql4, and Ddx11 colocalized with G4 structures more or less (Figure 2E). While in p53S cells, consistent with the Western blot results, we again observed higher level of Blm, Wrn, Recql4, and Ddx11 proteins than in p53null and WT cells, which colocalized and might attribute to the much less G4 structures in p53S cells (Figure 2E, p53S). Interestingly, in p53null cells, even with higher level of DNA helicase than in WT cells, the level of detectable G4 structures were also higher than in WT cells (Figure 2E, p53null and WT). These data indicated that p53S up-regulated the expression of DNA helicase and promoted the G4 unwinding by these helicases, which was not observed in p53null cells, suggesting this function as the gain of function of p53S mutant protein.

**Figure 3:**
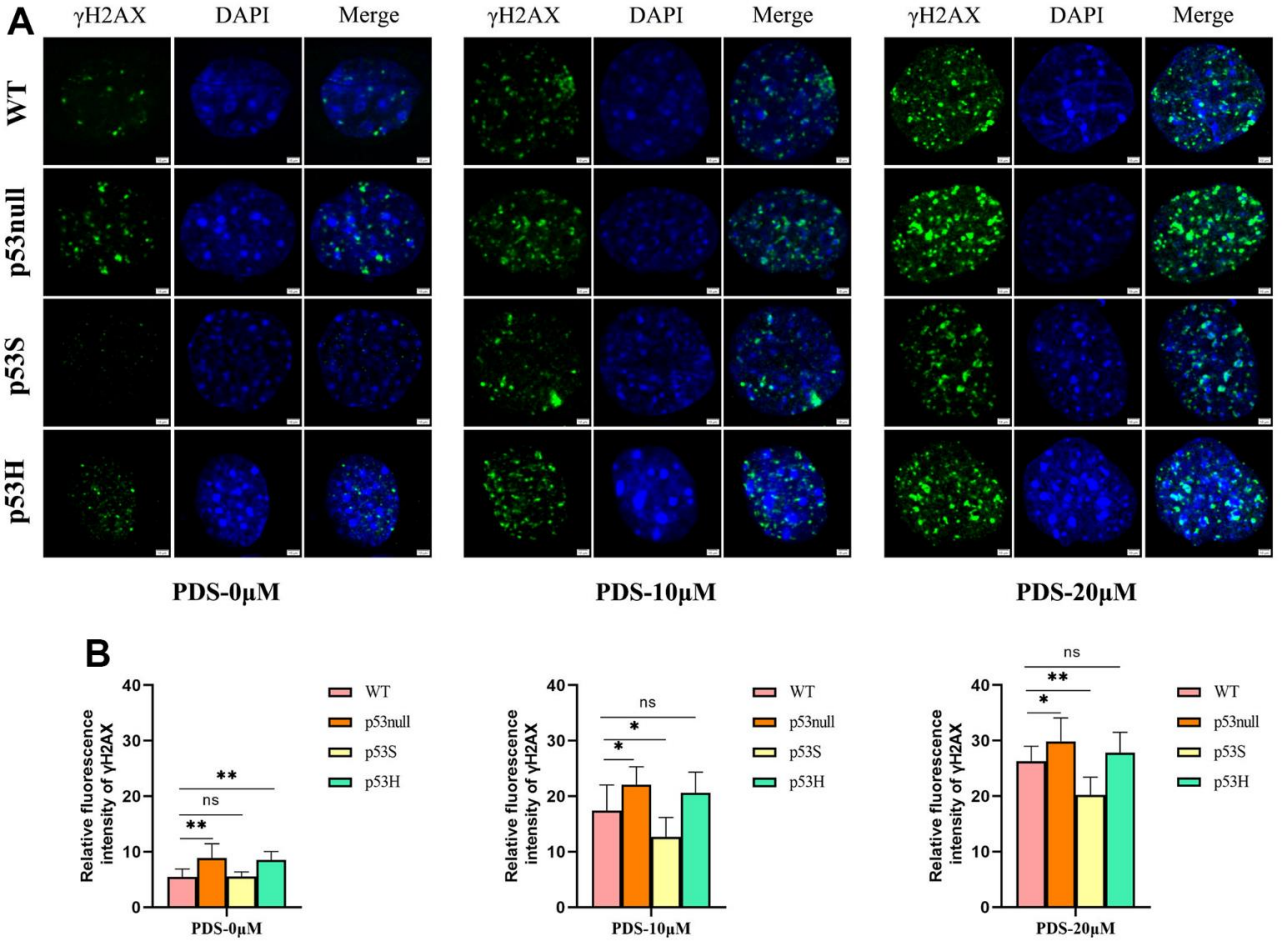
**The p53S protected the cells from G4 stabilizer PDS induced DNA damages.** (**A**) The presence of DNA damage related γ-H2AX foci induced by 10 μM or 20 μM of G4 stabilizer PDS in WT, p53null, p53S, and p53H cells. The p53null and the p53H cells showed stronger γ-H2AX signals compared with WT cells, and p53S cells displayed less γ-H2AX signals than WT cells. (**B**) The quantification of γ-H2AX signal in (**A**).

To further verify these data, we applied the G4 structure stabilizer pyridostatin (PDS) [27] to the WT, p53null, p53S, and p53H cells, and compared the cellular responses. By DNA damage marker γ-H2AX staining, we found that the p53S cells showed the least γ-H2AX signals upon the treatment of 10 μM and 20 μM PDS, while surprisingly, p53null and p53H cells showed much more abundant γ-H2AX signals than WT and p53S cells (Figure 3A). The amount of γ-H2AX signal was quantified in Figure 3B.

**Figure 4:**
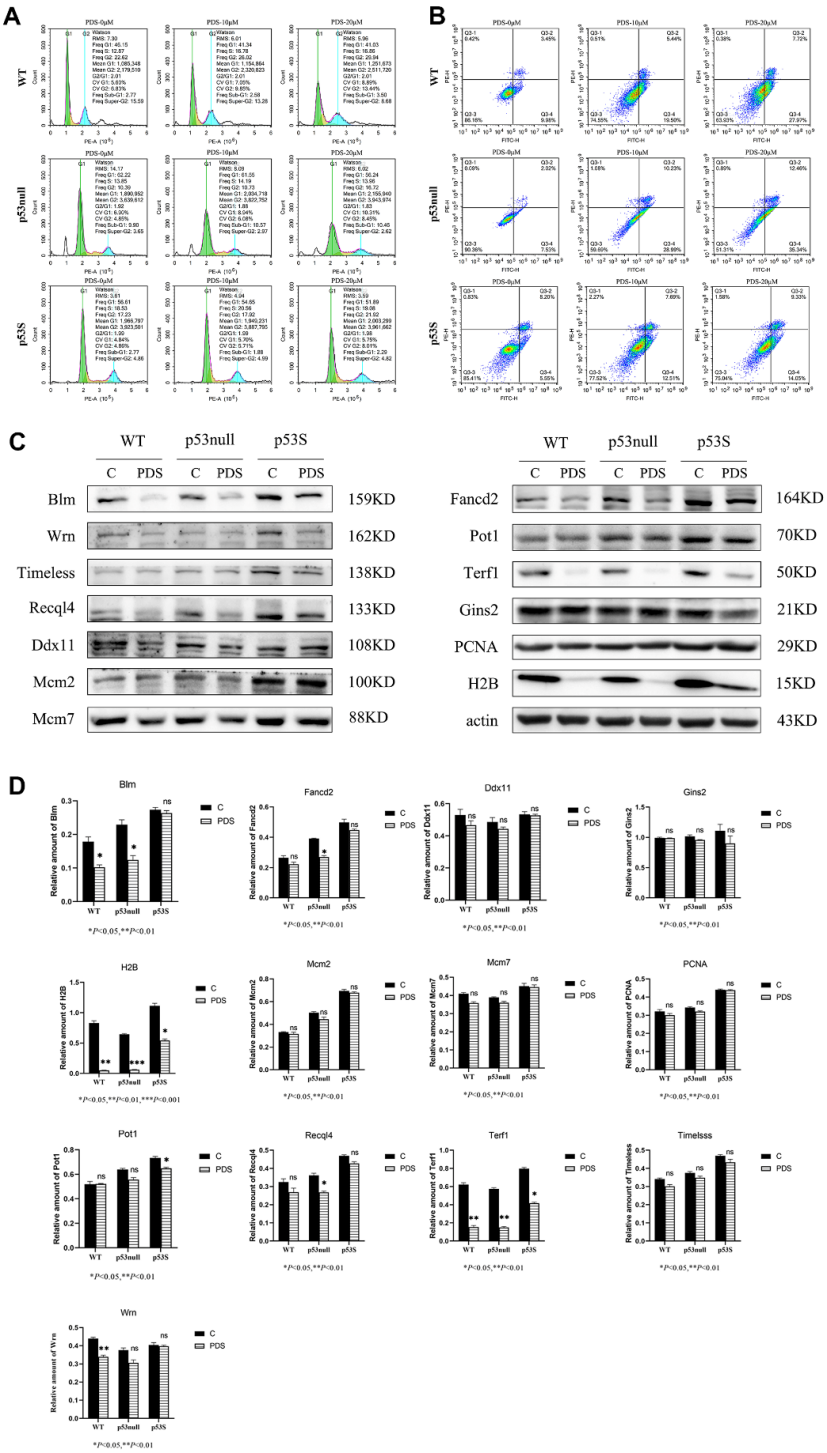
**The p53S endowed the cellular resistance to G4 stabilizer treatment.** (**A**) The cell cycle analysis revealed the G2 arrest induced by 20 μM PDS treatment, the p53null cells were most sensitive while the p53S cells were not sensitive. (**B**) The Annexin V staining displayed the cellular apoptosis induced by 20 μM PDS treatment. The p53null cells were most sensitive to PDS treatment, while p53S endowed the cell’s resistance to PDS treatment. (**C**) The DNA helicase protein level decreased after 20 μM PDS treatment, while p53S still maintained high level of DNA helicase. (**D**) The quantification of protein expression level in (**C**).

The cell cycle results revealed that upon the PDS treatment, the WT cells showed a slight increase of G2 phase in a dose-dependent manner, and a slight increase of sub-G1 phase when the PDS concentration was 20 μM (Figure 4A, WT), the p53null cells showed an obvious increase of G2 phase when treated with 20 μM PDS (Figure 4A, p53null), while the p53S cells showed only a slight increase of G2 phase even when the PDS concentration was 20 μM (Figure 4A, p53S).

By using Annexin V staining and flow cytometry assay, we found that apoptotic cells dramatically increased in both the WT and p53null cells upon PDS treatment in a dose-dependent manner (Figure 4B, WT, p53null). While in p53S cells, only a slight increase of apoptosis was detected (Figure 4B, p53S).

We further detected the DNA helicase protein level after PDS treatment. Again, we found that without treatment, the p53S cells expressed the higher protein level of DNA helicases than p53null and WT cells (Figure 4C, control). To our surprise, the expression of G4 structure related DNA helicase, such as Blm, Wrn, Timeless, Recql4 reduced obviously upon PDS treatment, though p53S cells still maintained the higher level of these DNA helicases compared to the p53null and WT cells. While the general DNA helicase, such as Mcm2, Mcm7, Gins2 were just slightly down-regulated. Interestingly, the telomere-related proteins Terf1 and Fancd2 also showed obvious down-regulation in p53null and WT cells upon PDS treatment, while Pot1 was just slightly down-regulated (Figure 4C, PDS). These data suggest that G4 stabilizer treatment resulted in the DNA replication stress and cell cycle arrest, specifically S-G2 arrest, which in turn feedback and down-regulated the DNA replication progress, surprisingly, the DNA helicases for resolving G4 structure were affected most.

Together these data revealed that the p53S protected the cells from DNA damages induced by G4 stabilizer PDS, and endowed the cells’ resistance to DNA replication stress, which might due to the high expression level of DNA helicases.

### The p53S promoted the DNA replication through the recruitment of helicases to the replication forks

From the above data, we speculate that the up-regulated helicases in p53S cells contributed to the initiation of DNA replication forks and unwinding of G4 structures, which make sure the fast progress of DNA replication with less replication stress. To further verify this, we modified the original iPOND assay [23] to detect the proteins connected with active DNA replication forks in cells.

**Figure 5:**
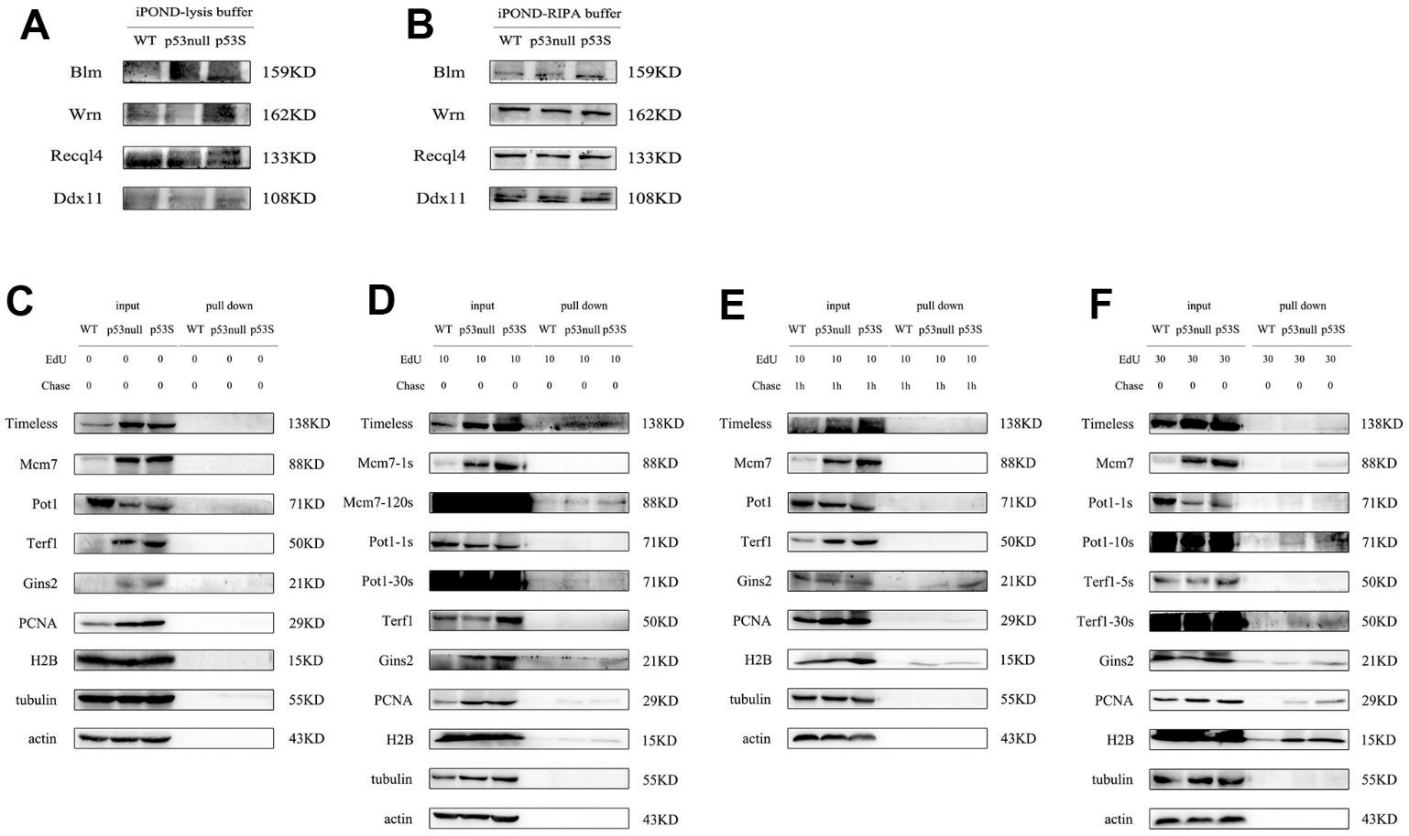
**The modified iPOND assay revealed that the p53S promoted the DNA replication through the recruitment of helicases to the replication forks.** (**A**) The resolution of high molecular weight proteins was not good with original iPOND assay. (**B**) The resolution of high molecular weight proteins was improved by the modified iPOND assay. (**C**–**F**) The DNA helicase and DNA fork binding proteins revealed by modified iPOND assay. (**C**) The control without EdU incorporation. (**D**) EdU incorporated for 10 min. (**E**) EdU incorporated for 10 min, followed by thymidine chase for 1h. (**F**) EdU incorporated for 30 min.

To compare the replication fork binding proteins from different type of cells, we need to give same amount of total protein. However, the lysis buffer of original iPOND assay contains 1% SDS, which affects the protein quantification by regular BCA assay. To solve this problem, we diluted the cell lysate to reduce the SDS concentration, and used Coomasie brilliant blue assay to do the protein quantification. Another problem for the original iPOND is the dissect of proteins with high molecular weight. We found that the high molecular weight proteins, such as Wrn, Blm, Recql4, Ddx11, were very hard to detect, the background was very high, and the bands were fuzzy (Figure 5A). We guessed this was due to the high SDS concentration in the cell lysate, we replaced the original lysis buffer with RIPA buffer, and re-did the iPOND assay, the resolution of those high molecular weight proteins was improved dramatically (Figure 5B).

With the iPOND assay, we analyzed the proteins bound to DNA replication forks. As the blank control, without EdU incorporation, we did not detect any non-specific binding (Figure 5C). By EdU incorporation for 10 min, we could already pull-down replication fork bound Timeless, as well as Mcm7, Gins2. And the p53S cell contained more replication fork bound Timeless, Mcm7, Gins2 proteins (Figure 5D). The telomere protein Pot1 was also detected in all three cells, without obvious difference (Figure 5D, Pot1). The positive control proteins PCNA and H2B could also been pulled down by 10 min EdU incorporation (Figure 5D, PCNA, H2B). We then did the 10 min EdU incorporation and followed by thymidine chase, to push the EdU labeled DNA away from the newly formed replication forks. After the thymidine chase, we could not detect Timeless, Mcm7, which confirmed that the Timeless, Mcm7 only bound to the replication forks and functioned there (Figure 5E). While we still could detect Gins2 and Pot1, suggested that they continuously bound to DNA strands (Figure 5E). We further did 30 min EdU incorporation, again we detected clearly the replication fork bound Timeless, Mcm7, Gins2, and Pot1, and they were more abundant in p53S cells (Figure 5F). Other than these, we could also detect small amount of Terf1 proteins bound to the replication forks, might reflect the replication forks in telomere DNA (Figure 5F, Terf1). However, we did not detect the specific G4 structure unwinding helicases, such as Wrn, Blm, Recql4 etc. located on the replication forks, even by EdU incorporation for 30 min, which suggested these helicases were not directly bound to the DNA replication forks with newly synthesized DNA.

Together these data revealed that the p53S promoted the recruitment of DNA helicase such as Timeless, Mcm2, Mcm7, Gins2 to the replication forks, which might facilitate the progress of replication forks, and reduced the replication stress.

### The p53S facilitated the telomere maintenance by reducing the telomere DNA replication stress

**Figure 6:**
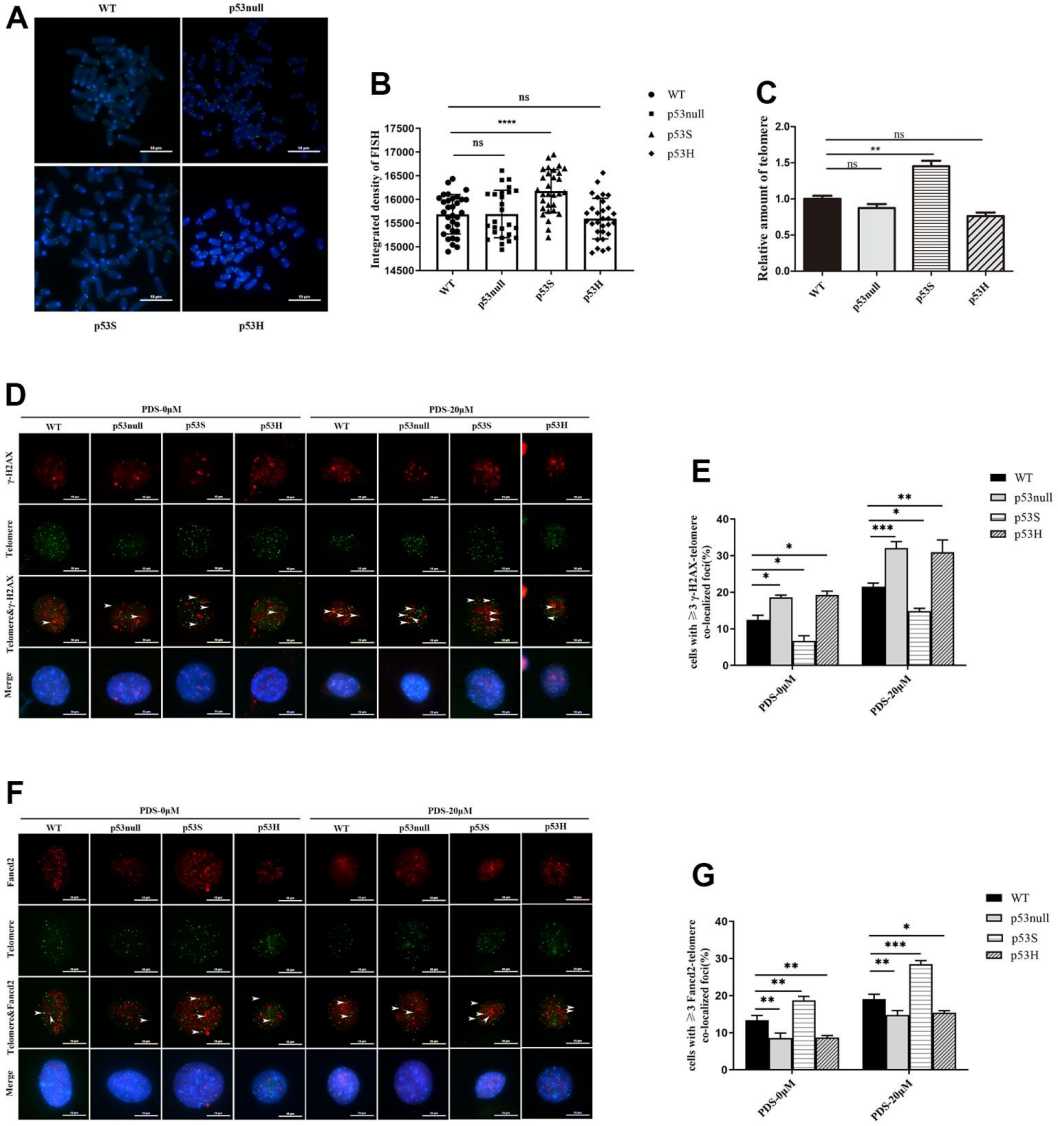
**The p53S facilitated the telomere maintenance by reducing the telomere DNA replication stress.** (**A**) The telomere FISH revealed the increased telomere length in p53S cells, but not in p53H cells. (**B**) The quantification and statistical analysis of (**A**). (**C**) The telomere DNA qPCR revealed the increased telomere length in p53S cells, but not in p53H cells. (**D**) IF-FISH revealed the increase of telomere DNA damage related γ-H2AX foci induced by 20 μM of PDS in all four cells, while the p53null and p53H cells showed stronger γ-H2AX signals compared with WT cells, and p53S cells displayed less γ-H2AX signals. The arrows pointed to the colocalization sites. (**E**) The quantification and statistical analysis of (**D**). (**F**) IF-FISH revealed that the telomere related DNA helicase Fand2 were recruited to the telomere after 20 μM PDS treatment, and p53S promoted the recruitment of Fancd2 to the telomere, but not in p53H cells. The arrows pointed to the colocalization sites. (**G**) The quantification and statistical analysis of (**F**).

From the above data, we were wondering whether the up-regulation of DNA helicases, as well as telomere proteins Terf1, Pot1, Fancd2, could impact the telomere maintenance in p53S cells. We measured the telomere length in WT, p53null, p53S, and p53H cells by qFISH (Figure 6A, 6B) and qPCR (Figure 6C). The results showed that telomere signals were significantly increased in p53S cells than that in WT and p53null cells (Figure 6A, 6B). The qPCR of telomere also showed the same result (Figure 6C). Interestingly, the p53H cells contained similar telomere signals with p53null cells detected by both qFISH (Figure 6A, 6B) and qPCR assay (Figure 6C). These data suggest that p53S facilitates the telomere maintenance, probably through the reduction of replication stress, and this effect is not observed in p53H mutation.

Given that Fancd2 has emerged recently as a key factor specially in regulating telomere DNA replication stress [28, 29], and the abundant G4 structures are the major source of DNA replication stress in telomere region. We used the G4 stabilizer PDS to induce DNA replication stress, and test the telomere DNA damages and the response of telomere protein Fancd2 in WT, p53null, p53S, and p53H cells.

Using γ-H2AX foci as DNA damage marker, we tested the telomere dysfunction induced foci (TIFs) in the indicated cells. Without PDS treatment, we observed the least background TIFs in p53S cells, and more in p53null and p53H cells (Figure 6D, 6E, PDS-0 μM). With 20 μM PDS treatment, we found a significant increase of DNA damage foci at telomeres in p53null, p53H and WT cells, while in p53S cells, the colocalization of γ-H2AX with telomere also increased, but was still the least among these cells (Figure 6D, 6E, PDS-20 μM).

We further evaluated the recruitment of Fancd2 at telomeres before and after PDS treatment. Without PDS treatment, we already observed more telomere located Fancd2 in p53S cells (Figure 6F, 6G, PDS-0 μM). After PDS treatment, we observed an increase of the amount of telomere located Fancd2 in all four cells, while the p53S still recruited more Fancd2 to the telomere DNA than p53null, p53H, or WT cells (Figure 6F, 6G, PDS-20 μM).

These data suggest that p53S facilitates the recruitment of Fancd2 on telomere DNA, and protected the telomere from DNA replication stress induced DNA damages.

## DISCUSSION

DNA replication stress has been one of the major obstacles for rapid cell proliferation. Understanding the cellular regulation and response to DNA replication stress is essential for both anti-aging and anti-tumor strategy. The p53S was a special anti-progeroid and tumorigenic p53 mutation we revealed earlier [20, 30]. However, it is not a common-sense hot spot mutation in human cancer, which results in the speculation of its actual biological significance in anti-aging.

The transcriptional and expression profile of p53S revealed the new gain of function of p53S in regulating cell cycle checkpoint, such as G2M, mitotic spindle [21]. To understand the underneath mechanism for these processes, here we further analyzed the detail pathways and genes regulated by p53S, and found that p53S could transcriptionally up-regulate the helicases expression, and recruit those helicases to the DNA replication forks, promote the unwinding of G4 structures, and ensure the smooth progression of DNA replication in the fragile chromosome sites. Thus p53S might promote DNA replication and telomere maintenance via regulating helicase function. On the other hand, the p53null cells also showed up-regulated DNA helicases, however, did not display any advantages in unwinding G4 structure, or protected cells from G4 stabilizer induced DNA replication stress. These data strongly support that the regulation of helicases in resolving G4 structure is the gain of function of p53S, which adds a new gain of function to mutant p53 family.

To test whether this gain of function is specific to p53S mutation, we utilized the MEFs bearing p53 hot spot mutation p53H from a mouse model of Li-Fraumeni Syndrome. Comparing the content of G4 structure, the replication stress induced by G4 stabilizer PDS, as well as the telomere lengthening in p53S and p53H cells, we found that only p53S, but not p53H, could facilitate the G4 structure resolving, and endow the cellular resistance to DNA damaged induced by G4 stabilizer. As the consequence, it is not surprising to observe that only p53S, but not p53H, could facilitate the lengthening of telomere. These data suggest that the gain of function in telomere lengthening may be specific to p53S mutation, via the regulation of G4 structure and replication stress.

Interestingly, as we observed before, the same p53S point mutation occurred in all three independent immortalized Werner syndrome MEFs with the mechanism of alternative lengthening of telomere [20, 31]. We were puzzled by the reason why p53S was the one that occurred in all three independent cell lines. Now this finding of the specific gain of function for G4 structure and telomere lengthening in p53S might explain the phenomenon. Telomere is the well-known difficult to replicate template. It is very interesting that we found p53S could up-regulated the expression level of telomere related protein Blm, Wrn, Timeless, Terf1, Pot1, and Fancd2, which might provide a new insight into the mechanism for telomere maintenance. Wrn and Blm are believed to share overlapped function and might somewhat compensate for each other in resolving telomere G4 structure [32]. Our data demonstrated that both Wrn and Blm were up-regulated and recruited to G4 structure in p53S cells, however, they were not been detected in DNA replication forks by iPOND assay. We proposed this might be due to the physical distance between nascent DNA and the G4 structure, new technique is required to link G4 and replication fork to reveal the proteins. It has been demonstrated that Timeless depletion caused increased levels of DNA damage, slowed telomere replication, and led to telomere aberrations [33]. Our data revealed that the increased Timeless proteins were recruited to the replication forks and promoted the replication progress, which might contribute to the telomere lengthening regulated by p53S.

The role of Terf1 and Terf2 on telomere DNA replication has been controversial. The single-molecule analysis of replicating telomeres has shown that Terf1 promoted efficient replication of TTAGGG repeats and prevents fork stalling [34]. Another study also showed that Terf1 recruited Blm to facilitate telomeric lagging strand DNA synthesis, and the Terf1 also deployed Tin2 and the Tpp1/Pot1 heterodimers in shelterin to prevent DNA damage induced ATR signaling during telomere replication [35]. While a study showed that Terf1 and Terf2 significantly stalled the replication fork progression at telomeric repeats [36]. Here we found that Terf1 was up-regulated in p53S cells, which gained longer telomere with less DNA replication stress on telomere.

We observed that most DNA replication related proteins showed highest expression level in p53S, and higher level in p53null than that in WT cells. We also observed that some proteins showed similar expression level in p53S and p53null, which was higher than WT cells. We guess this is due to protein specific regulation.

Here we also reported a useful modification for the iPOND technique. The well-known drawback of iPOND assay is the difficulty in detection of high molecular weight proteins, high background, fuzzy bands, etc. [37]. We replaced the original iPOND lysis buffer with RIPA buffer, reduced the SDS concentration and solved this problem. This modified iPOND assay could benefit the detection of high molecular weight proteins.

Many studies have shown that the aging process is the double-edged sword for tumorigenesis, and *vice versa*. We have speculated that the balance between the regulating factors for tumorigenesis and aging would be the cure for both tumor and progeroid disease.

It has been found that the cancer cells could benefit from overexpressing Claspin and Timeless to counteract the replication stress caused by oncogene-induced rapid firing of DNA replication [35]. We have also found that p53S could reduce the DNA damage responses caused by oncogenic H-Ras, and facilitate the tumorigenesis [20]. Here the role of p53S in regulating DNA helicase function explained how p53S helped to reduce the replication stress caused by fast firing of DNA replication by H-Ras signaling. Our data suggest the possible application of G4 stabilizer in the treatment of fast-growing tumors, especially tumors with abnormal oncogene activation and increased DNA replication firing.

Together our data suggest that p53S gains the new function of regulating DNA helicase, G4 structure, and releasing replication stress, that facilitate telomere lengthening. Interestingly, this gain of function may be specific to p53S mutation, which explains the function of p53S as driving force in the immortalization of senescent Werner syndrome cells. We are trying to screen natural compounds that could mimic p53S function, and rescue the aging phenotypes of progeroid syndrome via regulating helicase function and replication stress.

## MATERIALS AND METHODS

### Bioinformatics analysis

The ChIP-chip and RNA-seq experiments were done previously [21]. Here we applied the single sample gene set enrichment analysis (ssGSEA) [38] to further analyze the ChIP-chip data for DNA replication related pathways. The enrichment peaks in individual gene promoter were used as DNA binding affinity for ChIP-chip ssGSEA analysis. The C2 gene sets from Molecular Signatures database [39] were used as the pathway database for ssGSEA analysis. The gene set enrichment analysis (GSEA) [38] was applied on RNA-seq data to further analyze the individual genes involved in DNA replication pathways. The KEGG gene sets from Molecular Signatures database [39] were used as the pathway database for GSEA analysis.

### MEF cells and plasmids

The MEF cells of p53S (*p53^S/S^*), p53null (*p53^-/-^*), and WT (wild type) were harvested in 13.5 days and cultured in Dulbecco's Modified Eagle Medium (DMEM) with 10% fetal bovine serum (FBS) at 37° C with 5% CO_2_ and 3% O_2_. The p53H (*p53^H/H^*) MEF is kindly provided by Dr. Gigi Lozano from MD Anderson Cancer Center. Except p53H, all the MEFs were used for experiments before passage 5.

For G4 structure stabilizer treatment, the cultured cells were treated with 10 μM or 20 μM pyridostatin hydrochloride (PDS) (MCE) for 48h.

The G4 binding protein G4P was exogenously expressed in WT, p53null, p53S and p53H MEFs by transient transfection of pNLS-G4P-IRES2-EGFP plasmids [25], and the nuclear GFP fluorescence was used to estimate the amount of G4 structure.

### Western blot

Cells were harvested and lysed in RIPA buffer containing protease inhibitor cocktail (Roche, Switzerland). The 20 μg of total protein were separated by SDS-PAGE and then transferred to PVDF membrane. After blocking in 10% non-fat milk for 1 h at room temperature, membranes were incubated with primary antibodies overnight at 4° C. The membranes were then incubated with horseradish peroxidase-labeled secondary antibodies, and visualized with ECL. The primary antibodies used were anti-Blm (1:1000, Invitrogen, USA), anti-Wrn (1:1000, Invitrogen), anti-Recql4 (1:1000, Invitrogen), anti-Timeless (1:1000, Invitrogen), anti-Ddx11 (1:1000, Invitrogen), anti-Pot1 (1:1000, Invitrogen), anti-Fancd2 (1:1000, Novus Biologicals, USA), anti-Terf1 (1:1000, Invitrogen), anti-Gins2 (1:500, Santa Cruz, USA), anti-Top2a (1:1000, Novus Biologicals), anti-Mcm7 (1:1000, Santa Cruz), anti-Mcm2 (1:1000, Abcam, UK), anti-PCNA (1:1000, Abcam), anti-H2B (1:1000, Abcam), anti-tubulin (1:5000, Proteintech, USA), anti-actin (1:1000, Santa Cruz).

### Flow cytometry assays

For cell cycle analysis, cells treated with or without PDS were harvested and fixed in ice-cold 70% ethanol, stained with 1x PBS based propidium iodide solution (50 μg/ml PI, 100 μg/ml RNase A, 0.1% sodium citrate, 0.1% Triton X-100), and analyzed by flow cytometry (Agilent, USA).

For Annexin V staining, cells treated with or without PDS were harvested and stained with Annexin V-FITC and PI solution, and analyzed by flow cytometry (Agilent).

### Immunofluorescence

The cells cultured on cover slips were fixed with 2% paraformaldehyde and 2% sucrose in 1×PBS for 10 min and then permeabilized with 1% NP-40. After pre-incubation with 5% BSA/PBS, cells were incubated first with the primary antibody and then with the secondary antibody in 1% BSA/PBS for 1 h at room temperature. The slides were mounted with anti-fade mounting medium with DAPI (Solarbio, China). The primary antibodies used were anti-γH2AX (1:500, Abcam), anti-DNA G-quadruplex (G4) antibody, clone 1H6 (1:50, Merck, USA), anti-Blm (1:100, Invitrogen), anti-Wrn (1:100, Invitrogen), anti-Recql4 (1:100, Invitrogen), anti-Ddx11 (1:100, Invitrogen).

### Chromatin immunoprecipitation (ChIP) assay

The cell lysates from the wildtype (WT) and *p53^S/S^* MEFs were collected, and cross-linked by 1% formaldehyde, followed by sonication. After sonication, the supernatant was collected and pre-absorbed with ChIP grade protein A magnetic beads (Millipore, USA) and then incubated with the same beads together with anti-p53 antibody (Millipore) or IgG (GE Healthcare, USA) overnight at 4° C. The beads were then washed and eluted by elution buffer (1% SDS, 0.2M NaHCO3). Twenty microliters of 5M NaCl was added to the eluted product and incubated at 65° C for 4h to reverse the crosslinking. Immunoprecipitated genomic DNA was then purified using a QIAGEN Purification Kit for the semi-quantitative PCR.

### iPOND assay

The iPOND assay was applied according to the original methods [22]. Briefly, the cultured cells were labeled by 10 μM EdU for 10 min, 30 min, with or without 10 μM thymidine chase for 1h, followed by the fixation and crosslinking of DNA-protein by adding 10ml 1% formaldehyde solution in each flask for 20 min at room temperature. After adding 1ml 1.25M glycine to quench the crosslinking reaction, the cells were harvested and permeabilized for click reaction. The cells were then incubated in click reaction buffer containing 10 mM sodium ascorbate, 2 mM CuSO4, 10 μM biotin-azide, in PBS at room temperature for 2h. The EdU-Biotin labeled cells were then applied to lysis buffer containing 1% SDS in 50 mM Tris-HCl, pH 8.0, and proteinase inhibitor cocktail (Roche). The cell lysate was then sonicated and centrifuged, the supernatant was collected and diluted for further use. The input samples were taken at this step. For pulling down the Biotin labelled DNA-protein complex, the lysate was incubated with streptavidin-agarose beads (Millipore) overnight in dark. The captured complex was then applied for standard SDS-PAGE and Western blot.

The modification of original iPOND assay is the following: The lysis buffer of original iPOND assay contains 1% SDS, which affects the protein quantification by regular BCA assay. To solve this problem, we did a serial dilution (1:10, 1:100, 1:200, 1:400, 1:800, 1:1600) of the cell lysate by double distilled water to test the best ratio of dilution, and used Coomasie brilliant blue assay to do the protein quantification. Based on the standard curve made with same lysis buffer with same serial dilution, we found the 1:1000 as the best dilution ratio with accurate quantification and good read out. The Coomasie brilliant blue assay was used to quantify the protein concentration. The presence of high concentration of SDS also affects the SDS-PAGE effect, especially for proteins with high molecular weight. The background was high, and the protein band was not formed properly. We tried dialysis to reduce the amount of SDS, but this way wasted a lot of protein samples. We then used RIPA buffer (25 mM Tris-HCl pH 7.6, 150 mM NaCl, 1% NP-40, 1% sodium deoxycholate, 0.1% SDS) to replace the original lysis buffer. Compared to the original iPOND lysis buffer, the use of RIPA buffer provided similar protein purification, but better resolution, especially for high molecular weight proteins.

### Telomere assays

Immunofluorescence and fluorescent *in situ* hybridization (IF-FISH): Cells cultured on coverslips were treated with/without 20 μM PDS for 48 hours. After washing twice in 1×PBS, cells were fixed for 10 minutes in 2% sucrose and 2% paraformaldehyde, and permeabilized with 0.5% NP-40. After blocking in blocking solution (2% Gelatin and 0.5% BSA in 1×PBS), the primary antibody was applied overnight at 4° C and followed by secondary antibody incubation for 1h at room temperature in the dark. After washing three times in PBST, the cells were fixed with 4% paraformaldehyde for 10 minutes at room temperature, and hybridized with a telomere PNA-FISH probe 5′-FITC-green-(TTAGGG)-3′ (Panagene, South Korea). Coverslips were washed and counterstained with VECTASHIELD (Vector, USA). The primary antibodies for IF-FISH: γ-H2AX (1:500, Millipore), Fancd2 (1:500, Novus Biologicals). A minimum of 200 nuclei for each cell type were analyzed for colocalization with telomeres. The p-values were calculated by two-way ANOVA analysis followed by Tukey’s multiple comparison.

Telomere PNA-FISH: Cells were treated with 200ng/mL of Colchicine (Meilunbio, China) for 4 hours before harvest. Chromosomes were fixed with 4% formamide and hybridized with a telomere PNA-FISH probe 5′-FITC-green-(TTAGGG)-3′ (Panagene) for 3h at room temperature in the dark. After washing, the coverslips were counterstained with VECTASHIELD (Vector). The relative telomere signals were analyzed with ImageJ. A minimum of 200 nuclei for each cell type were analyzed for colocalization with telomeres. The p-values were calculated by two-way ANOVA analysis followed by Tukey’s multiple comparison.

Telomere DNA qPCR assay: The cultured cells were harvested and the genomic DNA was purified for Syber green based real time PCR. The telomere DNA primer sequences were: Forward: 5’-CGGTTTGTTTGGGTTTGGGTTTGGGTTTGGGTTTGGGTT-3’, Reverse: 5’-GGCTTGCCTTACCCTTACCCTTACCCTTACCCTTACCCT-3’. The reference 36B4 DNA primer sequences were: Forward: 5’-ACTGGTCTAGGACCCGAGAAG-3’, Reverse: 5’- TCAATGGTGCCTCTGGAGATT-3’.

## Supplementary Material

Supplementary Figure 1
