# Supplementary material for: Modified iPOND revealed the role of mutant p53 in promoting helicase function and telomere maintenance
Source: Aging (Albany NY). 2023 Oct 12;15(19):10767–84. doi: 10.18632/aging.205117 (PMC10599736; doi:10.18632/aging.205117)
Supplement: Supplementary Figure 1 [file aging-15-205117-s001.pdf]

SUPPLEMENTARY FIGURE

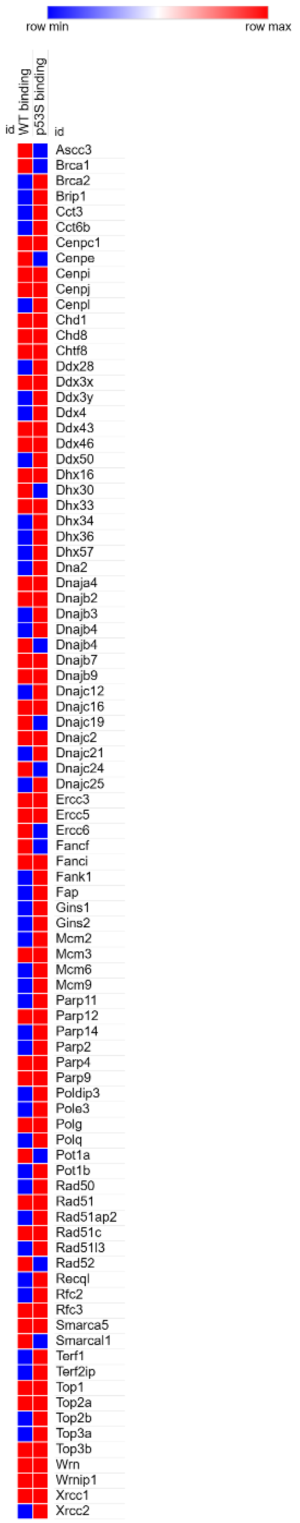

Supplementary Figure 1. The heatmap of genes involved in DNA replication pathways and DNA helicase pathways shown in Figure 1A.
